# Nanomaterial enabled sensors for environmental contaminants

**DOI:** 10.1186/s12951-018-0419-1

**Published:** 2018-11-22

**Authors:** Marjorie R. Willner, Peter J. Vikesland

**Affiliations:** Department of Civil and Environmental Engineering and the Institute for Critical Technology and Applied Science, Center for Sustainable Nanotechnology (VTSuN), Virginia Tech, Blacksburg, USA

**Keywords:** Nanomaterials, Sensor, Detection, Environment, Pesticides, Heavy metals, Pathogens

## Abstract

The need and desire to understand the environment, especially the quality of one’s local water and air, has continued to expand with the emergence of the digital age. The bottleneck in understanding the environment has switched from being able to store all of the data collected to collecting enough data on a broad range of contaminants of environmental concern. Nanomaterial enabled sensors represent a suite of technologies developed over the last 15 years for the highly specific and sensitive detection of environmental contaminants. With the promise of facile, low cost, field-deployable technology, the ability to quantitatively understand nature in a systematic way will soon be a reality. In this review, we first introduce nanosensor design before exploring the application of nanosensors for the detection of three classes of environmental contaminants: pesticides, heavy metals, and pathogens.

## Background

Nanomaterial enabled sensors are an exciting technology that provide exquisite detection, on the nanomolar to sub-picomolar level, of environmental contaminants [1–5]. Interest in these sensors stems from their potential for facile, in-field contaminant detection without the need for expensive lab equipment. Many past reviews in this area have grouped sensors based on the signal transduction method [2–5], nanoparticle backbone [7–10], or contaminant class [1, 11, 12], thus leaving one important paradigm virtually untouched: classifying sensors based on the analyte(s) of interest. Because environmental scientists and engineers are often interested in determining if a specific contaminant exists at a field site and if its concentration is above the regulatory limit, there was a need to organize a review based upon the detection of specific contaminants. This review has been developed to address these concerns. First, we summarize the general concepts underlying a nano-enabled sensor and then discuss recent developments in nanomaterial enabled detection of nine specific analytes: two pesticides, four metals, and three pathogens. A nearly infinite number of chemicals of environmental concern exist and although it would be impossible to outline all of them, the fundamental nanosensor designs can be seen in the examples outlined within the review. For the reader interested in nanosensors for pharmaceutical detection we direct them to the work of Nagaraj et al. [13] and the reviews of Sanvicens et al. [14] and Cristea et al. on antibiotic detection [15].

## Introduction

**Fig. 1:**
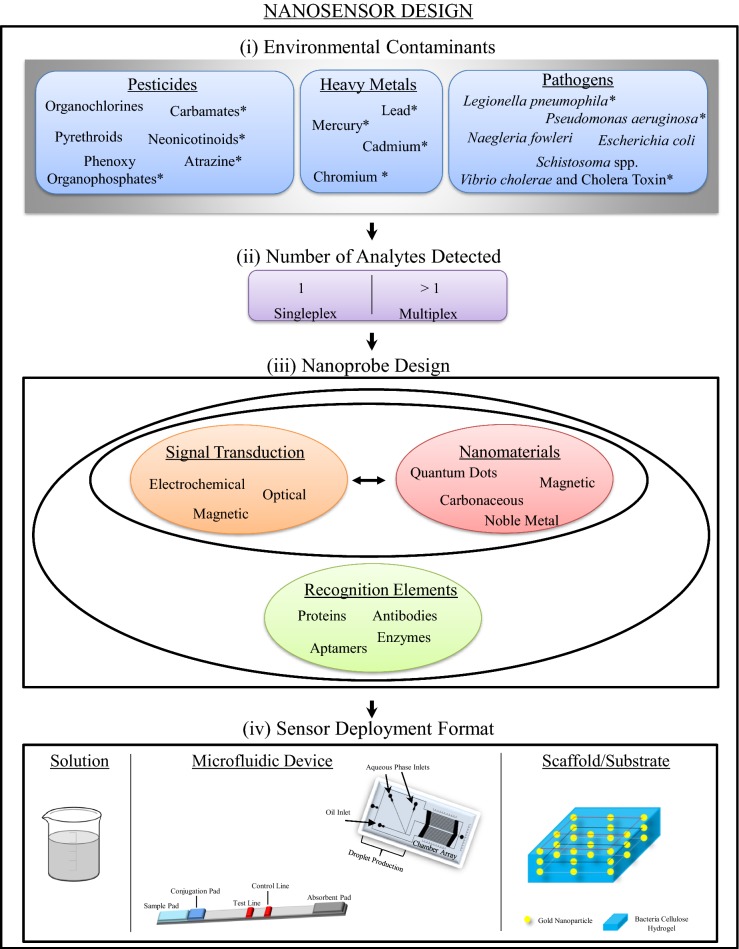
Nanosensor design schematic. First, a class and subsequently a specific contaminant of interest is selected (i). The contaminants discussed in this review are denoted with an asterisk. Next, the number of analytes to be detected by the sensor is chosen (ii) and then the probe is designed. A nanoprobe consists of two core elements, a signal transduction method and at least one nanomaterial, and may also include a recognition element (iii). Ultimately, the sensor deployment format is selected (iv)

Nanomaterial enabled sensors consist of three components: a nanomaterial(s), a recognition element that provides specificity, and a signal transduction method that provides a means of relaying the presence of the analyte (Fig. 1). These components are not necessarily distinct entities within a sensor, but every nanosensor can be characterized on the basis of these three divisions. Sensors can be designed to detect a single analyte or multiple analytes, termed multiplex detection. In addition to detecting an analyte by producing a signal, a ‘turn-on’ or ‘off/on’ sensor, some of the sensors described below are based on a ‘turn-off’ or ‘on/off’ mechanism, where-by a decrease in signal indicates the presence of an analyte.

### Nanomaterials

Nanomaterials have enabled advances in sensor design such as miniaturization, portability, and rapid signal response times. High surface area to volume ratios and facile surface functionalization make nanomaterials highly sensitive to changes in surface chemistry thus enabling nanosensors to achieve extremely low detection limits. In some cases, the enhanced sensitivity of nano-enabled sensors is due to the fact that nanomaterials are of a similar size as the analyte of interest (e.g., metal ions, pathogens, biomolecules, antibodies, DNA) and are thus capable of interrogating previously unreachable matrices [4]. We briefly introduce three different general nanomaterial classes: quantum dots (QDs), metal nanoparticles, and carbonaceous nanomaterials.

#### Quantum dots

QDs are semiconductor nanocrystals with a typical composition *MX* where *M* is commonly cadmium (Cd) or zinc (Zn) and *X* is selenium (Se), sulfur (S), or tellurium (Te). QDs are often coated by a second *MX* alloy, a shell, to create core/shell QDs with highly tuned properties. Common QDs employed in sensor applications include: CdSe [16], CdSe/ZnS [17–19], CdTe [20–25], CdTe/CdS [22], ZnS [26], and ZnSe/ZnS [27]. QDs have characteristically narrow fluorescence emission bands, yet broad absorption bands, thus making them excellent optical transducers. Moreover, QD emission wavelengths can be readily adjusted by changing the size, shape or composition of the QD. Accordingly, QDs are ideal for multiplex detection of a number of different analytes. QDs diverse in shape, size, and composition can be excited by a single energy source because they have broad absorption spectra.

#### Metal and metal oxide nanoparticles

Because of their capacity to be produced in a wide variety of shapes, their high extinction coefficients (ε > 3 × 10^11^ M^−1^ cm^−1^) [28], and their facile surface functionalization, noble metal nanoparticles (NP) have been extensively used in a number of sensor applications. Colloidal solutions of gold and silver nanoparticles, AuNP and AgNP respectively, exhibit unique colors based on the size of the colloidal nanomaterial. For example, AuNP spheres in the ~ 5 to ~ 50 nm diameter range appear red in color but become more purple in hue as they increase in size towards ~ 100 nm. This color change can be exploited for use in visual colorimetric sensors where the presence of an analyte causes small nanoparticles to aggregate and the solution to change color. Gold and silver nanoparticle excitation can lead to the uniform oscillation of conduction electrons. This uniform oscillation gives rise to localized surface plasmon resonance (LSPR) [29] based spectroscopies such as surface plasmon resonance (SPR) and surface enhanced Raman spectroscopy (SERS). Plasmon based spectroscopies are discussed in greater detail below and elsewhere [28–30].

The chemistry of metal NPs, particularly AuNPs, has been exploited for use in highly selective sensors [31, 32]. We note that although it is possible to use AgNPs for sensor applications, the anti-microbial activity of silver [33] and its propensity to dissolve often limits the utility of such sensors. Gold NPs are stable, biocompatible, and have been extensively explored for use in sensing applications [7]. Surface coatings can be used to modify the particles and facilitate the attachment of recognition elements. Thiol capping agents provide colloidal stability and chemical functionality. Two commonly used thiols are thioglycolic acid (TGA) and 3-mercaptopropionic acid (MPA). These two agents impart a negative surface charge and create nanoparticles with an extremely high colloidal stability [34]. The choice of capping agent depends on the desired function and nanoparticle composition. The interested reader is referred to recent reviews by Saha et al. [7] and Wei et al. [35] for additional details on gold enabled sensors.

A range of nanostructured metal oxides (NMOs) have been explored for sensing applications. NMOs include: iron oxides, titanium oxides, zirconium oxides, cerium oxides, zinc oxides, and tin oxides. Magnetic iron oxides, such as magnetite (Fe_3_O_4_) and maghemite (γ-Fe_3_O_4_), have low toxicity, are economically friendly, and can be easily functionalized with ligands, antibodies, and other capping agents [36]. One important allure of magnetic NPs arises from their use in facilitated separation processes and remediation applications [12]. Titanium dioxide, TiO_2_, has also been embraced in nano-sensor design [37, 38], but it is most typically used and studied for its photocatalytic properties.

#### Carbon-based nanomaterials

Carbon nanotubes (CNTs) and graphene are often employed in nano-enabled sensors because of their large surface area, excellent electrical conductivity, high thermal conductivity and mechanical strength [39]. One recent application of these nanomaterials has been their use to increase the sensitivity of glassy carbon electrodes (GCE) for electrochemical sensing [40, 41]. Other sensor designs have exploited the electronic properties of graphene for fluorescence quenching. In such a design, as discussed later in this review, a QD with a recognition element is conjugated to a graphene sheet and in the presence of the analyte the sensor undergoes a conformational change that separates the QD from the graphene and “turns-on” the sensor.

### Recognition elements

Selectivity is an extremely important facet in the design of a successful biosensor. A diverse array of recognition elements have been implemented in nanosensor design including antibodies [42–46], aptamers [47–52], enzymes [53], and functional proteins [54]. The two most widely used agents, antibodies and aptamers, are described here in detail.

#### Antibodies

Antibodies (Abs) are proteins produced by the immune system in response to foreign agents [55]. They exhibit highly specific binding to a single antigen and are widely used in the capture and labeling of microorganisms and other materials that elicit an immune response [56]. Three types of antibodies have been used for analyte recognition: polyclonal (pAbs), monoclonal (mAbs), and engineered antibody fragments [57]. While antibodies are widely used in biosensors, there are a number of drawbacks to antibody use that include: high development costs, temperature and pH sensitivity, batch-to-batch variation, and short shelf-lives [44, 58]. Despite these disadvantages, for immunogenic analytes (i.e., those that elicit an immune response) Abs are often the most selective recognition agent [59]. Sensors that incorporate antibodies, either one type or multiple, are commonly referred to as immunosensors or immunoassays. A common descriptor of an electrochemical immunosensor is “label-free” because changes in the properties of the transducer surface owing to the antibody-antigen interaction can be directly measured [60].

#### Aptamers

Aptamers are flexible short oligonucleotide strands, either RNA or single stranded DNA (ssDNA), used to bind specific molecules. Produced both naturally and synthetically, aptamers have been designed to recognize toxic and non-immunogenic substances [61]. Aptamer production is estimated to cost approximately 10–50× less than antibody production [62]. Additionally, aptamers have low batch-to-batch variability, long shelf-lives, and are thermally stable [63]. Nucleic acid aptamers can be synthesized de novo with high specificity due either to the use of the systematic evolution of ligands by exponential enrichment (SELEX) process [64] or other newer isolation and synthesis approaches [65]. The SELEX process starts out by exposing a large library (> 10^14^ strands) of random oligonucleotide sequences to the target sequence. Through affinity testing and polymerase chain reaction (PCR) amplification the oligonucleotide sequences with the tightest binding are isolated, their sequences determined, and following de novo synthesis can be incorporated into biosensors.

### Signal transduction

The three major signal transduction methods employed in nano-enabled sensors are optical, electrochemical, and magnetic. Optical techniques, particularly colorimetric sensors that report a signal in the visible spectrum, are desirable for wide-scale use by the general public. A well-known example of a colorimetric biosensor is the home pregnancy test. Electrochemical sensing methods have high specificity and can be simplistic and facile to miniaturize [2]. Compared with optical and electrochemical methods, magnetic transduction methods exhibit minimal background signal thus making them ideal for low concentration samples. Other sensor designs use magnetic materials to pre-concentrate the analyte prior to use of an optical or electrochemical transduction method.

#### Optical

Optical transduction is based on the interaction of a sensing element with electromagnetic radiation. Analytical techniques monitor emission or absorption of a sample under irradiation by ultraviolet, visible, or infrared light [66]. Two common optical methods utilized in nanosensor design are fluorescence and surface plasmon resonance enabled spectroscopies.

Fluorescence spectroscopy is based upon measurement of the emission of a fluorophore as it returns to its ground state following excitation. Fluorescent nanosensor applications often employ QDs or dye-doped silicon or polymer nanoparticle probes because they are photostable and are generally more robust than traditional fluorescent dyes [67, 68]. Designs are described by the change in the fluorescence signal upon interaction with an analyte of interest as either “turn-off” or “turn-on”. Quenching or restoration of the fluorescence signal may be a result of a direct interaction between the analyte and the nanoparticle or a conformational change in the sensor.

Surface plasmon resonance enabled spectroscopies are an optical transduction technique based on the localized surface plasmon resonance (LSPR) of noble metal nanomaterials [3, 35]. The LSPR band is sensitive to the mean interparticle distance and therefore can be used to observe changes from a dispersed to an aggregated system or vice versa. Commonly, the LSPR is used in conjugation with a secondary spectroscopy technique to create a surface enhanced spectroscopy: surface enhanced fluorescence (SEF) or surface enhanced Raman spectroscopy (SERS) [67].

#### Electrochemical

Electrochemical detection methods measure the change in current or potential that result from the interaction between an analyte and an electrode. A multitude of techniques have been used to observe these changes and include cyclic voltammetry, chronoamperometry, chronopotentiometry, impedance spectroscopy, and various field-effect transistor based methods [4]. Nano-enabled sensor designs can involve modification of the solid electrode (e.g., platinum, gold, silver, graphite) with nano-carbons (e.g., carbon nanotubes, graphene) or functionalization with recognition elements (e.g., antibodies, aptamers) [2].

Direct spatial contact between the nanoscale architecture of the electrode and the recognition element gives rise to large signal amplification and improved signal to noise ratios compared to traditional electrochemical techniques [2, 4, 69]. In addition to the electrode properties, the size and morphology of the analyte of interest has been shown to affect sensor function. Improved detection limits have been shown for smaller particles due to their higher diffusivity and lower steric hindrance [70].

#### Magnetic

Magnetic transduction has been embraced for detection in biological samples because of the low background magnetic signal [71] and the fact that magnetic nanoparticles (MNP) can be collected under an applied magnetic field regardless of the optical properties of the solution [67]. Often, the use of magnetic nanoparticles to concentrate, separate and purify the analyte of interest in the detection zone is termed magnetic transduction [71]. However, a secondary transduction method, such as electrochemical stripping, can often be employed and therefore use of the term magnetic transduction can be a misnomer.

Magnetic-relaxation switches that incorporate superparamagnetic iron oxide nanoparticles are a pure form of magnetic transduction. The principle underlying this detection mechanism is the clustering of individual nanomagnetic probes into larger assemblies following interaction with a target. Analyte binding results in the formation of NP clusters and enhanced dephasing of the spins of the surrounding water protons. The subsequent change in the spin–spin (T2) relaxation can be detected by magnetic resonance relaxometry [9, 72]. Magnetic relaxation switches have been used to detect nucleic acids (DNA and mRNA), proteins [73] and viruses [74] among other targets.

## Analytes

As defined at the outset of this review, a wide variety of different analytes can be detected by nanomaterial-based sensors. In this portion of the review, we focus explicitly on the applications of nanosensors towards detection of pesticides, metals, and pathogens.

### Pesticides

There is great interest in detection of pesticides given their widespread use, their toxicity, and their proclivity for bioaccumulation. Currently, over 800 active ingredients, in 100 different substance classes are present in commercial pesticides [75]; we summarize the major pesticide classes in Table 1. Organophosphorus (OP), carbamates, neonicotinoids, and triazines are the dominant classes and to date have been the focus of nano-enabled pesticide detection. Liu et al. [75], Verma et al. [76], Aragay et al. [1], Evtugyn et al. [60] and Pang et al. [77], provide detailed reviews of pesticide detection techniques. In this section, a brief background on pesticide detection will be followed by a discussion of recent advances.

**Table 1:** Common pesticide classes

Class of chemical pesticides	Examples	Types	Effects
Carbamates	Carbaryl, methomyl, propoxur, aldicarb	Fungicide, insecticide, acaricide	Non-persistent, cholinesterase-inhibiting, not very selective, toxic to birds and fish
Neonicotinoids	Acetamiprid, clothianidin, imidacloprid, nitenpyram, nithiazine, thiacloprid, thiamethoxam	Insecticide	Water soluble, concern regarding persistence and bioaccumulation
Organochlorines	Aldrin, chlordane, dieldrin, endrin, heptachlor; lindane, methoxychlor; toxaphene, hexachlorobenzene (HCB), pentachlorophenol (PCP), DDT	Insecticide, acaricide, fungicide	Persistent, bioaccumulative, affects the ability to reproduce, develop, and to withstand environmental stress by depressing the nervous, endocrine and immune systems
Organophosphates	Schradan; parathion; malathion	Insecticide, acaricide	Non-persistent, systemic (cholinesterase-inhibiting), not very selective, toxic to humans
Phenoxy	2,4-D and 2,4,5-T	Herbicide	Selective effects on humans and mammals are not well known 2,4-D: potential to cause cancer in laboratory animals 2,4,5-T: is the source of a toxic contaminant dioxin
Pyrethroids	Fenpropathrin, deltamethrin, cypermethrin	Insecticide	Target-specific -more selective than the organophosphates or carbamates, generally not acutely toxic to birds or mammals but particularly toxic to aquatic species
Triazines	Atrazine, cyanazine, and simazine	Herbicides	Persistent, binds to the plastoquinone-binding protein in photosystem II, endocrine disruptor in humans

#### Organophosphates

Pesticides are often designed to impact a specific enzyme; many forms of pesticide detection are based on observing and monitoring this enzyme either directly or indirectly. Organophosphate and carbamate pesticides inhibit the production of acetylcholinesterase (AChE) an enzyme that catalyzes the hydrolysis of acetylcholine, a neurotransmitter [78, 79]. The fundamental reaction is shown in Eq. 1.

1 $$acetycholine + {\text{H}}_{2} {\text{O}} \mathop \to \limits^{AChE} choline + acetate.$$

A class of rapid and sensitive electrochemical sensors has been developed around the immobilization of AChE on a solid electrode surface [41, 80–82]. The products of Eq. 1 are not electroactive, and thus to detect the inhibition of AChE an analogous reaction based on the hydrolysis of acetylthiocholine is typically used [83].

**Fig. 2:**
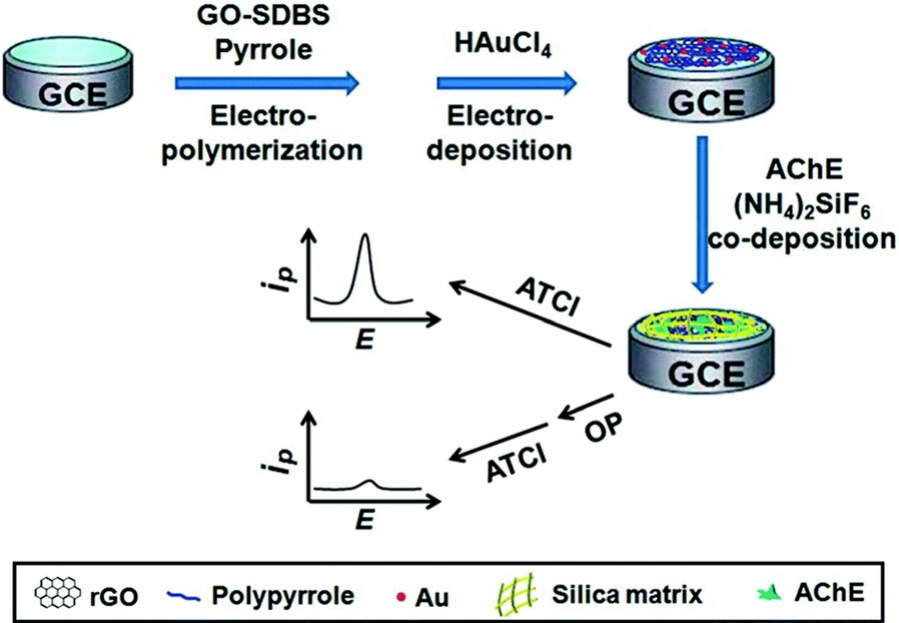
Illustration of the preparation of the Au–PPy–rGO nanocomposite-based AChE biosensor. Illustration of the preparation of the Au–PPy–rGO nanocomposite-based AChE biosensor and its application for the electrochemical detection of organophosphorus pesticides (Reproduced from Yang et al. [84] with permission of The Royal Society of Chemistry)

For example, Yang et al. [84] combined two different types of nanomaterials, reduced graphene oxide (rGO) and gold nanoparticles, to achieve a detection limit of 0.5 nM for the model organophosphate paraoxon-ethyl (Fig. 2). Reduced graphene oxide sheets provide an increased surface area for AChE immobilization and were deposited with polypyrrole (PPy) to prevent aggregation. Gold nanoparticles (~ 20 nm) were then electrodeposited onto the PPy-rGO surface to further increase both the surface area and the conductivity of the electrode. The final step was co-deposition of AChE and a silica matrix, (NH_4_)_2_SiF_6_. The biocompatible silica matrix prevented the AChE from leaking out of the electrode and ensured that the enzymes maintained their bioactivity. The completed sensor was tested using cyclic voltammetry and AChE inhibition was defined based on the peak experimental current and control current.

Similarly, Yu et al. [85] used the large surface area of carbon nanotubes to create a sensitive organophosphorus pesticide (OP) biosensor. Amino functionalized carbon nanotubes (CNT–NH_2_) were dried on the surface of a standard glassy carbon electrode (GCE) and subsequently incubated with AChE. Using differential pulse voltammetry (DPV), the limit of detection of the CNT decorated GCE was 0.08 nM.

Recently, Cui et al. [86] reported the use of a nanocomposite to improve the stability of AChE electrochemical biosensors. A layer of reduced graphene oxide (rGO) was introduced onto a glassy carbon electrode, followed by deposition of a titanium dioxide (TiO_2_) porous sol–gel film mixed with chitosan (CS), a bio-compatible polymer. The stability of the matrix was further improved by the electro-deposition of a second layer of CS to yield a multi-layer mesoporous nanostructure. Total detection time required approximately 25 min and the limit of detection of dichlorvos, a model OP, was 29 nM. Although, the limit of detection of the sensor described in Yu et al. was better, without a side-to-side comparison of the sensors using the same test matrix no conclusion can be drawn with regard to sensor performance.

Pang et al. [87] explored the application of an aptamer SERS sensor in complex food samples. The assay utilized a unique aptamer developed by Zhang et al. [88] that can detect four distinct organophosphorous pesticides: phorate, profenofos, isocarbophos, and omethoate. Dendritic silver, an organized nanostructure, was selected as the SERS substrate because it provides locally consistent SERS enhancement factors [89]. The surface was decorated with aptamers and also a blocking agent, 6-mercaptohexanol (MH), to eliminate non-specific binding on the silver surface. Probes were incubated with the pesticides, removed from solution via centrifuge and dried prior to Raman interrogation. Analysis of each molecule’s unique Raman fingerprint led to the determination of four distinct limits of detection: phorate 0.4 μM, isocarbophos 3.5 μM, omethoate 24 μM, and profenofos 14 μM.

Recently, Nie et al. [90] reported a similar SERS-aptamer sensor, but with aqueous sample detection. Unlike Pang et al.’s requirement to wash and drop-dry the probes onto a glass slide, Nie et al. mixed a malathion specific antibody with positively charged spermine coated silver nanoparticles and directly collected SERS spectra from the suspension. The phosphate backbone of the aptamer is negatively charged and electrostatic interactions led the aptamer complex to attach to the silver nanoprobes.

Fewer reports have described traditional optical immunoassays, such as the lateral flow immunoassay (LFIA), for OP detection. Wang et al. [91] developed a “bare-eye” assay with antibody functionalized gold nanoparticles that enabled the user to visually verify the presence or absence of three pesticides of interest: two OPs, chlorpyrifos-methyl and isocarbophos, and imidacloprid, a neonicotinoid. Of the three antibodies used, the antibody for isocarbophos (neonicotinoid) had to be developed in-house because it had not previously been reported in the literature. In fact, antibodies exist for only about ~ 10% of the 800 active pesticide ingredients [75]. The production of a large library of pesticide antibodies has been stymied by the costs and difficulties in creating antibodies for these low molecular weight and non-rigid molecules [1].

#### Neonicotinoids

**Fig. 3:**
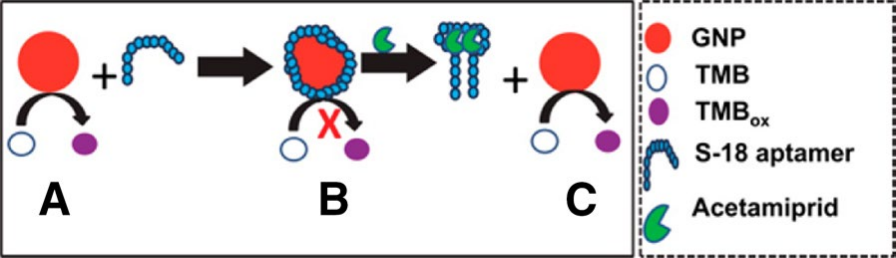
Schematic representation of acetamiprid detection. Schematic representation of the reversible inhibition of the nanozyme activity of GNPs using an acetamiprid-specific S-18 ssDNA aptamer. Step A shows intrinsic peroxidase-like activity of GNPs that gets inhibited after shielding of the GNP surface through conjugation of S-18 aptamer molecules (step B). In the presence of acetamiprid target, the aptamer undergoes target-responsive structural changes and forms a supramolecular complex with acetamiprid resulting in free GNP to resume its peroxidase like activity (step C) (Reprinted with permission from Weerathunge et al. [95]. Copyright 2014 American Chemical Society)

A class of neuro-active insecticides, neonicotinoids were first introduced in the 1980s and are currently the largest class of insecticides in use [92]. However, there are growing concerns regarding the impact of neonicotinoid to human health [93]. Nanosensors for neonicotinoid detection have focused specifically on the detection of acetamiprid with aptamers being the preferred recognition element as underscored by Verdian’s recent review paper [94]. For example, Weerathunge et al. [95] exploited standard aptamer functionality to create a novel sensor based on the peroxidase-like activity of gold nanoparticles (GNP). As shown in Fig. 3, the colorless reporter molecule 3,3,5,5-tetramethylbenzidine (TMB), which turns purplish-blue upon oxidation, was used to create an off/on sensor with a signal observable via UV–visible absorbance. In the presence of an acetamiprid-specific aptamer, the oxidation of TMB is blocked. The introduction of the target molecule led to the desorption of the aptamer and restoration of TMB oxidation within 10 min. The authors reported a limit of detection of 0.1 ppm (450 nM) with a dynamic linear detection range of 0.1–10 ppm.

#### Triazine

A class of nitrogen heterocycles, triazine detection is typically limited to atrazine detection because it is one of the most comonly used herbicides in the United States [96]. A range of label-based [97, 98] and label-free [99–101] designs have been embraced for the detection of atrazine. For example, Liu et al. [97] designed a competitive electrochemical immunoassay. A gold electrode decorated with gold nanoparticles was functionalized with anti-atrazine monoclonal antibodies. Differential pulse voltammetry measurements were then used to directly measure changes in the electrode surface resulting from the antibody-antigen interaction. The sensor was determined to be highly sensitive with a limit of detection of 74 pM.

**Fig. 4:**
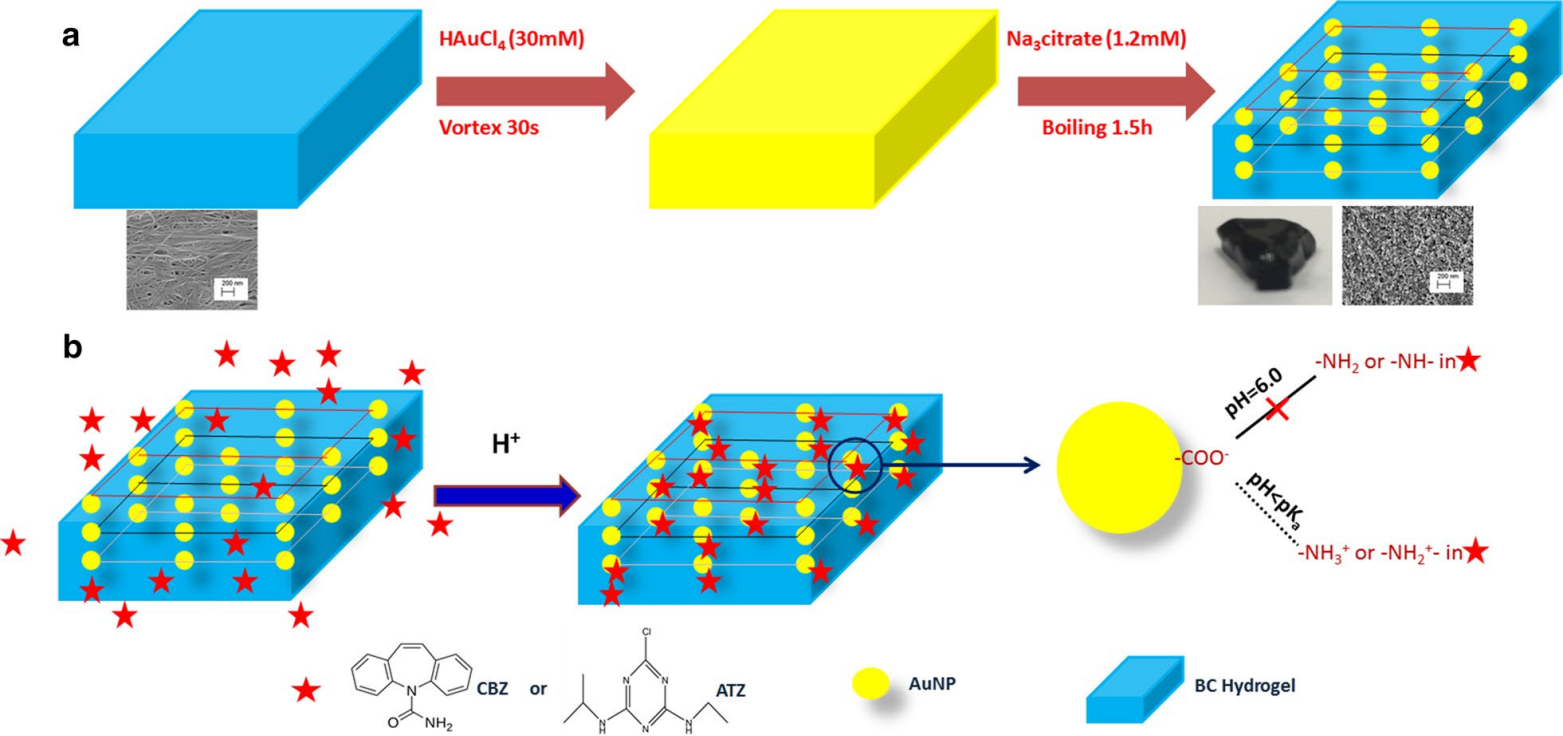
Schematic of synthesis of gold nanoparticle/bacteria cellulose nanocomposites and their applications. Schematic of synthesis of gold nanoparticle/bacteria cellulose nanocomposites (**a**). Schematic of pH-induced adsorption of carbamazepine (CBZ) and atrazine (ATZ) on AuNP/BC (**b**) (Reprinted with permission from Wei and Vikesland [99] from Springer Nature)

A unique label-free methods for atrazine detection was described by Wei and Vikesland [99]. A gold nanoparticle/bacteria cellulose (AuNP/BC) plasmonic nanocomposite was synthesized by the in situ reduction of gold salt in the presence of bacteria cellulose. As shown in Fig. 4, pH-triggered attachment of atrazine to the nanocomposite was achieved by lowering the pH of the solution below atrazine’s pK_a_ of 1.7 and was confirmed by an increase in the SERS signal in the AuNP/BC. Ultimately, the group was able to achieve a limit of detection of 11 nM, which is below the EPA’s maximum concentration of 3 μg/L for drinking water, but three orders of magnitude greater than the label-based detection.

### Metals

Nano-enabled sensors have been successfully developed for a number of heavy metals and in this section, we review mercury, lead, cadmium, and chromium detection. A diverse array of transducers and nanoparticles are used to detect these environmentally relevant contaminants all with the aim of developing sensitive and selective sensors. Readers interested in additional information about nanosensors for heavy metal detection are directed to the reviews of Li et al. [11] and Ullah et al. [102].

#### Mercury

**Fig. 5:**
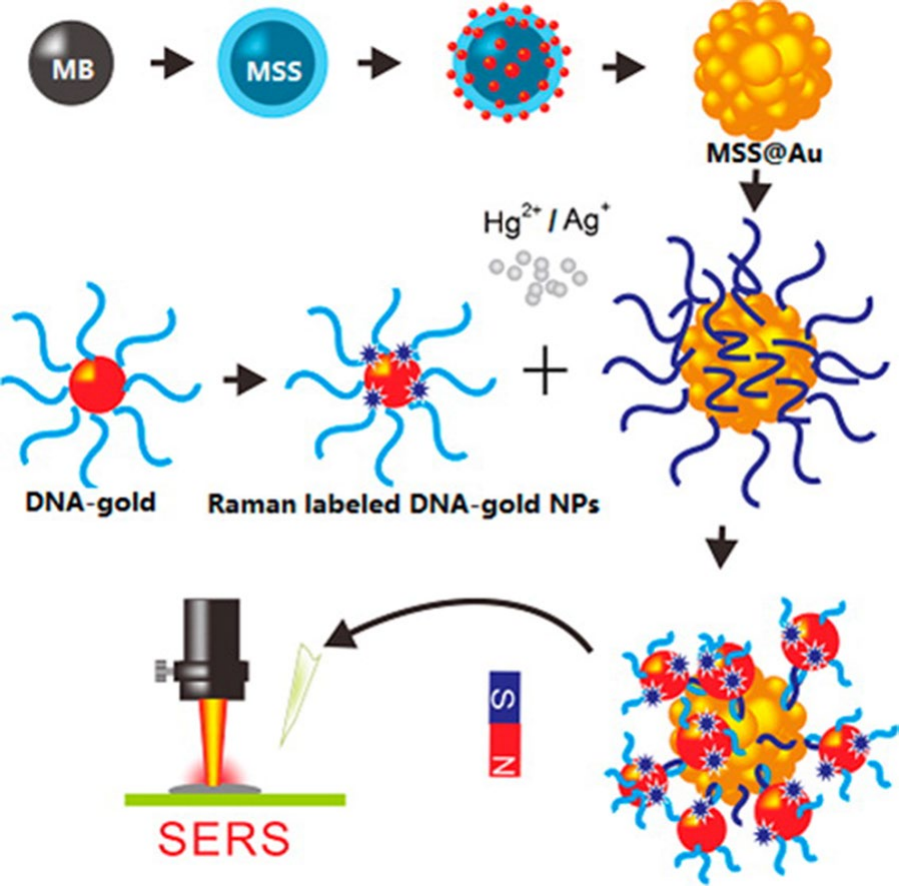
Schematic of SERS-active system for Hg^II^ ion detection. Schematic illustration of the SERS-active system for Hg^II^ ion detection based on T–Hg–T bridges using DNA-Au NPs and DNA-MSS@Au NPs (Reprinted with permission from Liu et al. [50]. Copyright 2014 American Chemical Society)

The negative neurological effects of mercury exposure to humans have driven extensive investigation into the geochemical cycling and detection of this element [103]. A major focus of mercury (Hg^II^) nanosensor development has been the production of DNA-based probes [47–50, 104–106]. Thymine–thymine (T–T) base-mismatches in DNA are significantly stabilized in the presence of Hg^II^ [104] due to the formation of metal base pairs [107]. Two major types of oligonucleotide mercury probes have been reported in the literature: G-quadruplexes [48, 49], which unfold, and nearly complementary single strands, which hybridize [106]. A growing number of mercury sensors are being constructed using multiple nano-elements, such as the mercury sandwich assay described by Liu et al. [50]. In this assay, magnetic silica spheres encapsulated in a gold shell and Raman labeled gold nanoparticles were functionalized with complementary DNA sequences that contained five mismatched thymine sites, Fig. 5. The DNA sequences were chosen such that the binding energy between the complementary aspects of the strands was insufficient to allow them to fully hybridize. In the presence of mercury, full hybridization occurred thus decreasing the inter-probe spacing and creating a plasmonic hotspot. Owing to the magnetic particle cores, the nanoprobes could be easily recovered with an external magnet and subsequently recycled.

Thiol mediated assays for mercury detection have been described in the literature for a variety of nanoparticles such as gold [108–111], silver [112] or quantum dots [27]. Aggregation [108] or disaggregation [109] are typically utilized to provide a colorimetric response. Reaction based competition assays in which Hg^II^ replaces a surface coating have also been described in the literature [32, 110]. Huang and Chang [110] created an on-sensor that emitted a fluorescence signal in the presence of mercury due to the displacement of rhodamine 6G (R6G) from the nanoparticle surface. In the process of iterating through three sensor designs to create a sensitive and selective assay, the authors found that thiol coatings increased the specificity of the assay for mercury. The final sensor was reported to have a limit of detection of 2.0 ppb and a rapid analysis time (< 10 min).

#### Lead

Associated with increased risk of cancer and subtle cognitive and neurological deficits [113], lead (Pb) is a heavy metal contaminant of major concern. Labeled and label-free nanosensors have both been reported for sensitive Pb^II^ detection. For label-based detection, the recognition element 8–17 DNAzyme, a catalytic nucleic acid, has been used [114, 115] as well as a class of oligonucleotides that form G-quadruplexes in the presence of lead [17, 48, 116].

Tang et al. [115] combined 8–17 DNAzyme with rolling circle amplification (RCA) and quantum dots to develop an electrochemical sensor with a limit of detection of 7.8 pM. In this assay, DNAzyme catalytic strands were immobilized onto a magnetic bead (MB) and then hybridized with a substrate strand containing a single sessile ribonucleoside adenosine (rA) to form double stranded DNA with a single stranded loop to accommodate Pb^II^ ion. In the presence of Pb^II^, the DNAzyme was activated to cleave the substrate strand at the rA group. The exposed single DNA strand, tethered to the MB, then hybridizes with the RCA template. Polymerase and deoxyribonucleotide triphosphates (dNTPs) were then added to trigger the RCA process and yield a long single stranded product with repeating sequence units. The complement of the RCA sequence was functionalized to CdS quantum dots leading to the hybridization of multiple QDs in a periodic arrangement. QD rich DNA duplexes were then magnetically separated from the solution and dissolved in nitric acid. The released cadmium cations were quantified via square wave voltammetry.

Gao et al. [117] developed an AlOOH-graphene oxide nanocomposite for the detection of lead and cadmium by square wave anodic stripping voltammetry (SWASV). In this assay, the fast electron transfer kinetics achieved with graphene oxide were coupled to the high adsorption capacity of AlOOH to create a nanocomposite with a LOD of 76 pM. Unlike the RCA method, the AlOOH was not selective for a single metal. However, since each metal has a unique stripping peak the AlOOH-graphene oxide nanocomposite could be used for multiplex detection.

#### Cadmium

The body of work on nano-enabled sensors for cadmium (Cd) detection is less robust than that for mercury and lead, but detection limits on the order of nano-molar have been reported. A variety of nanomaterials have been explored including QDs [22, 118], single wall carbon nanotubes (SWCNT) [119], and antimony nanoparticles [120].

Gui et al. [22] described an off/on-sensor fluorescence sensor for Cd^II^ detection. Photoluminescent CdTe/CdS QDs were first quenched (i.e., turned-off), by ammonium pyrrolidine dithiocarbamate (APDC) due to the partial loss of the Cd–thiol surface layer and subsequent surface passivation. Introduced cadmium ions displaced the APDC from the QD surface and restored the photoluminescence (PL); thus, turning the sensor on. The sensor was highly selective for Cd^II^, a threefold increase was seen in the PL intensity, and a limit of detection of 6 nM was determined.

Gui et al. [118] enhanced the accuracy of their Cd^II^ detection device by creating a ratiometric sensor. In this sensor, the fluorescence of two different chromophores was measured in order to minimize the error introduced by fluctuation in the photoluminescence of the QDs. To limit interactions between the QDs and the secondary dye, the CdTe QD cores were coated with a polymer, polyethylenimine (PEI), prior to conjugation with fluorescein isothiocyanate (FITC). The QDs were then quenched using sulfur (S^2−^) while the FITC signal was maintained. Again, upon introduction of cadmium the sensor was turned on and the photoluminescence was restored. The limit of detection was slightly higher for this sensor compared to the same groups initial report, 12 nM vs. 6 nM, but was linear across a much larger range, 0.1–15 µM compared with 0.1–2 µM.

#### Chromium

High chromium (Cr) absorption in vivo can result in various diseases, including fibro-proliferative diseases, airway hypersensitivity, lung cancer, nasal cancer, and other types of tumors [121]. Multiple immunoassays have been described for the detection of chromium [45, 46], but they are all based on the work of Liu et al. [46]. In pursuit of an immunochromatographic assay (ICA), Liu et al. developed novel anti-Cr^III^-EDTA monoclonal antibodies (McAb). Chromium ions are too small to elicit an immune response and thus they were mixed with the highly effective bifunctional chelating agent, isothiocyanobenzyl-EDTA, and conjugated to the carrier protein bovine serum albumin (BSA) before being introduced to mice from which the antibodies were ultimately extracted. The immunoassay dipstick was composed of the three main parts: (i) a conjugation pad that was dosed with the anti-Cr-EDTA antibodies; (ii) a test line that contained the analyte of interest, Cr-EDTA, and; (iii) a control line that contained goat- anti-mouse antibodies. To run a sample, liquid is introduced to the dipstick and travels into the conjugation pad where the probes are brought into solution. For a negative sample, the free antibody probes bind to the test line, whereas in a positive sample no probes will bind as all antibody sites are already occupied and thus no signal is produced at the test line. The antibodies at the control line will capture any probes in the solution even those that are bound to the target of interest and is use to verify that capillary action wicked the solution through the whole length of the dipstick. The ultimate result of Liu et al. was an assay with a visual limit of detection of 50 ng/mL and an analysis time of < 5 min.

### Pathogens

Ever since John Snow’s 1854 revelation that cholera was spread through the consumption of contaminated water, waterborne pathogen detection has been a key area of research. The World Health Organization (WHO) recognizes twelve bacteria, eight viruses, seven protozoa, and two helminths as pathogens of significance in drinking water supplies, as outlined in Table 2 [6]. Pathogen detection methods typically focus on: (i) whole analyte (cell) detection or detection of a representative epitope on the cell membrane; (ii) genetic material detection; or (iii) pathogenic product (e.g., toxin) detection. For the sake of brevity, herein we confine our discussion to the detection of *Vibrio cholerae* and the toxin it produces, cholera toxin, *Legionella pneumophila*, which was responsible for greater than 50% of the waterborne disease outbreaks between 2011 and 2012 [122], and *Pseudomonas aeruginosa,* which the WHO recently classified as a critical pathogen in light of the proliferation of antimicrobial resistant species [123]. For expanded reviews we refer the reader to the works of Kumar et al. [124] and Mocan et al. [125].

**Table 2:** Waterborne pathogens and their significance in water supplies Adapted from WHO Table 7.1 waterborne pathogens and their significance in water supplies [6]

Pathogen	Health significance	Persistence in water supplies
Bacteria		
*Burkholderia pseudomallei*	High	May multiply
*Campylobacter jejuni*, *C. coli*	High	Moderate
*Escherichia coli*—pathogenic	High	Moderate
*E. coli*—enterohaemorrhagic	High	Moderate
*Legionella* spp.	High	May multiply
Non-tuberculous mycobacteria	Low	May multiply
*Pseudomonas aeruginosa*	Moderate	May multiply
*Salmonella typhi*	High	Moderate
Other salmonellae	High	May multiply
*Shigella* spp.	High	Short
*Vibrio cholerae*	High	Short to long
*Yersinia enterocolitica*	Moderate	Long
Viruses		
Adenoviruses	Moderate	Long
Enteroviruses	High	Long
Astroviruses	Moderate	Long
Hepatitis A virus	High	Long
Hepatitis E virus	High	Long
Noroviruses	High	Long
Sapoviruses	High	Long
Rotavirus	High	Long
Protozoa		
*Acanthamoeba* spp.	High	May multiply
*Cryptosporidium parvum*	High	Long
*Cyclospora cayetanensis*	High	Long
*Entamoeba histolytica*	High	Moderate
*Giardia intestinalis*	High	Moderate
*Naegleria fowleri*	High	May multiply
*Toxoplasma gondii*	High	Long
Helminths		
*Dracunculus medinensis*	High	Moderate
*Schistosoma* spp.	High	Short

#### $Vibrio\; cholerae$ and cholera toxin

Cholera, the infamous disease that spawned germ theory is now virtually unknown in the United States, but it continues to pose a major disease burden around the world with an estimated 1.3–4.0 million cases of cholera a year leading to between 21,000 and 143,000 deaths [126]. Cholera is an acute diarrhoeal disease caused by the ingestion of contaminated water or food containing the bacterium *Vibrio cholerae*. In the intestines, the bacteria colonize the mucosa and begin to secrete cholera toxin (CT), which initiates the disease symptoms [127]. Nanosensors have been fabricated to detect both *Vibrio cholerae* [128, 129] and CT, but the majority of the literature has focused on detection of CT subunit B (CT-B) [130–134] because the subunit induces cellular uptake of the toxin and not all *V. cholerae* isolates are toxigenic [135]. Label-based detection of CT can be achieved using antibodies, ganglioside GM1 (the binding site of CT), or β-galactose, a sugar with strong affinity towards CT. Ahn et al. [130] provide a nice summary of CT-B detection and reported a fluorescence resonance energy transfer (FRET) based method with a theoretical detection limit of 280 pM. In FRET, fluorescence from QDs is quenched, and the energy is transferred by another particle such as a gold nanoparticle. The quenching is inhibited in the presence of the target. Specifically, the cholera toxin binds to the β-galactose modified gold nanoparticles prohibiting the binding of the QDs.

#### $Legionella\; pneumophila$

Named for the famous 1976 outbreak at the American Legion, Legionnaires’ disease is a pneumonia like disease caused by the bacterium *Legionella pneumophila*. Under specific conditions, the bacterium can flourish in building (premise) plumping and infect people when they inhale aerosols containing the infective agent. Two approaches have been presented in the literature for nano-enabled *Legionella* detection: whole organism detection [136, 137] and DNA detection [138–140].

Martin el al. [136] developed a whole organism sensor that combined a sandwich immunoassay for bacterial capture with amperometric transduction. Magnetic nanoparticles were modified with poly(dopamine) (pDA) and ultimately functionalized with specific capture antibodies (C-Ab) to create MNPs@pDA-C-Ab probes. After incubation with the sample, a second detector antibody labeled with horseradish peroxidase was introduced and a magnetic field was used to capture the immunocomplexes on a screen-printed carbon electrode (SPCE). The authors found the assay to be specific for *Legionella*, but they needed a preconcentration step in order to achieve a LOD below the reference of 100 colony-forming units (CFU) L^−1^. However, with a runtime of < 3 h, compared to 10 days for the standard approach, and a LOD of 10 CFU mL^−1,^ the sensor has the potential to be used as a rapid first screening method for highly contaminated water systems.

**Fig. 6:**
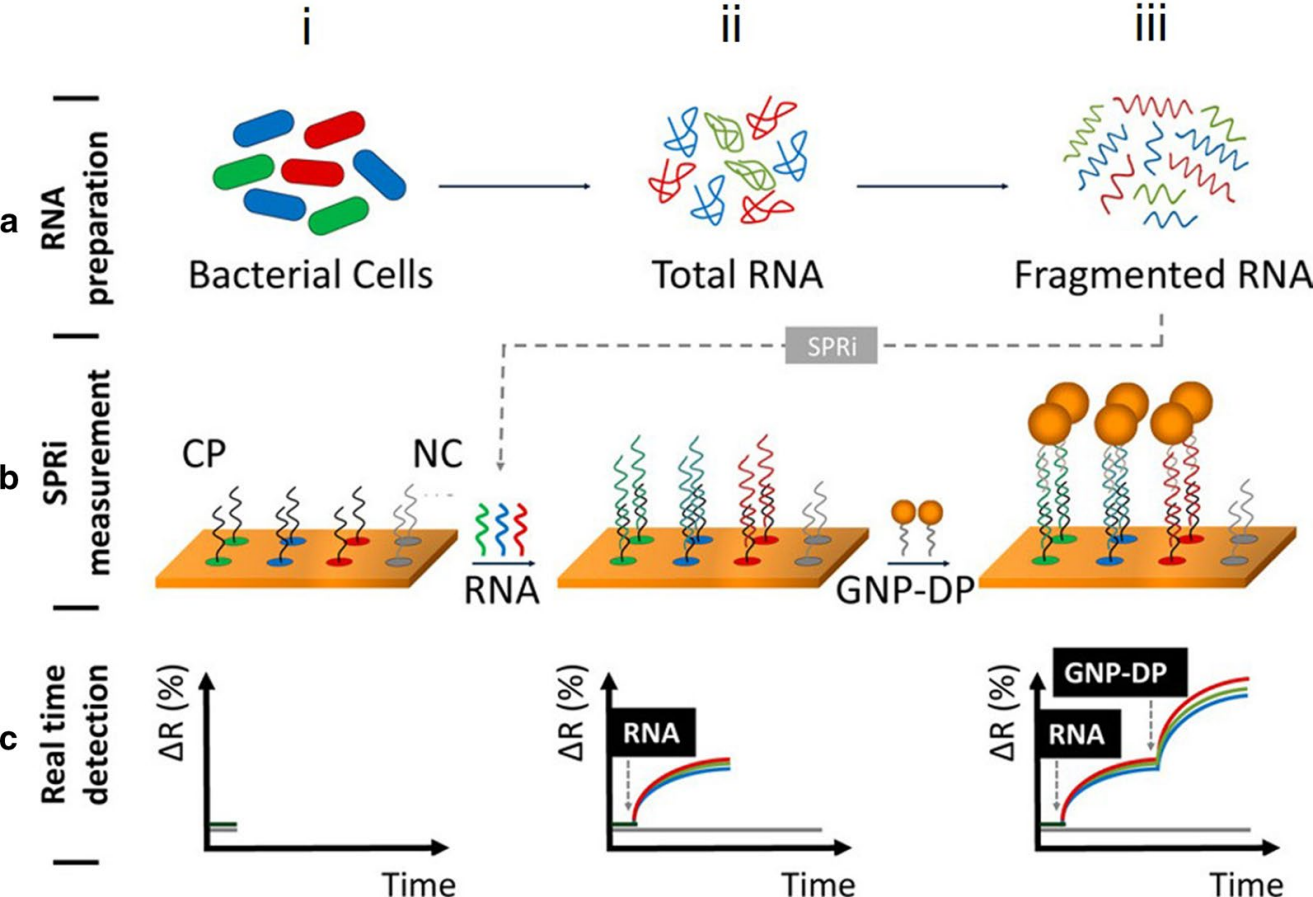
A schematic of multiplex RNA detection using surface plasmon resonance imaging (SPRi). A schematic of multiplex RNA detection using surface plasmon resonance imaging (SPRi). RNA fragments are first extracted from bacteria of interest (**a**). A biochip functionalized with three specific capture probes (CP) and a negative control probe (NP), each demarcated in a unique color (**b** (i)) is shown to exhibit no change in reflectivity (**c** (i)). Upon introduction to the RNA (**b** (ii)), there is an increase in single (**c** (ii)). Finally, gold nanoparticles functionalized with the detection probe (GNP-DP) are introduced and shown to enhance the change in reflectivity (Adapted with permission from Melaine et al. [139]. Copyright 2017 American Chemical Society)

In a recent report, Melaine et al. [139] described the multiplex detection of 16S rRNA from *Legionella*, *Pseudomonas aeruginosa* (discussed below) and *Salmonella typhimurium*. A DNA microarray with capture DNA specific for each target was assembled on a surface plasmon resonance imaging (SPRi) substrate, e.g., gold coated nanoprisms. Upon hybridization of the DNA with isolated 16S rRNA a change in the reflectivity signal was observed, as shown in the bottom of Fig. 6. To extend the dynamic range of detection and enhance sensitivity, gold nanoparticles functionalized with a detection probe were introduced to the sample and ultimately RNA at concentrations as low as 10 pg mL^−1^ were detected.

#### $Pseudomonas\; aeruginosa$

An opportunistic pathogen, *Pseudomonas aeruginosa* can be found in sources such as feces, soil, water, and sewage with the most important route of exposure being skin (dermal) contact with contaminated water or tools. Similar to *Legionella*, *P. aeruginosa* can colonize premise plumbing and has been associated with outbreaks of nosocomial infections in hospitals [141]. Most of the detection schemes reported for *P. aeruginosa* focus on whole pathogen detection [142–146] with the work of Melanie et al. [139], discussed above, on 16s rRNA detection being an outlier. In addition, to oligonucleotide recognition elements [139, 142–144], antibodies [145, 147] and bacteriophages [146] have also been used for specific detection of *P. aeruginosa*.

**Fig. 7:**
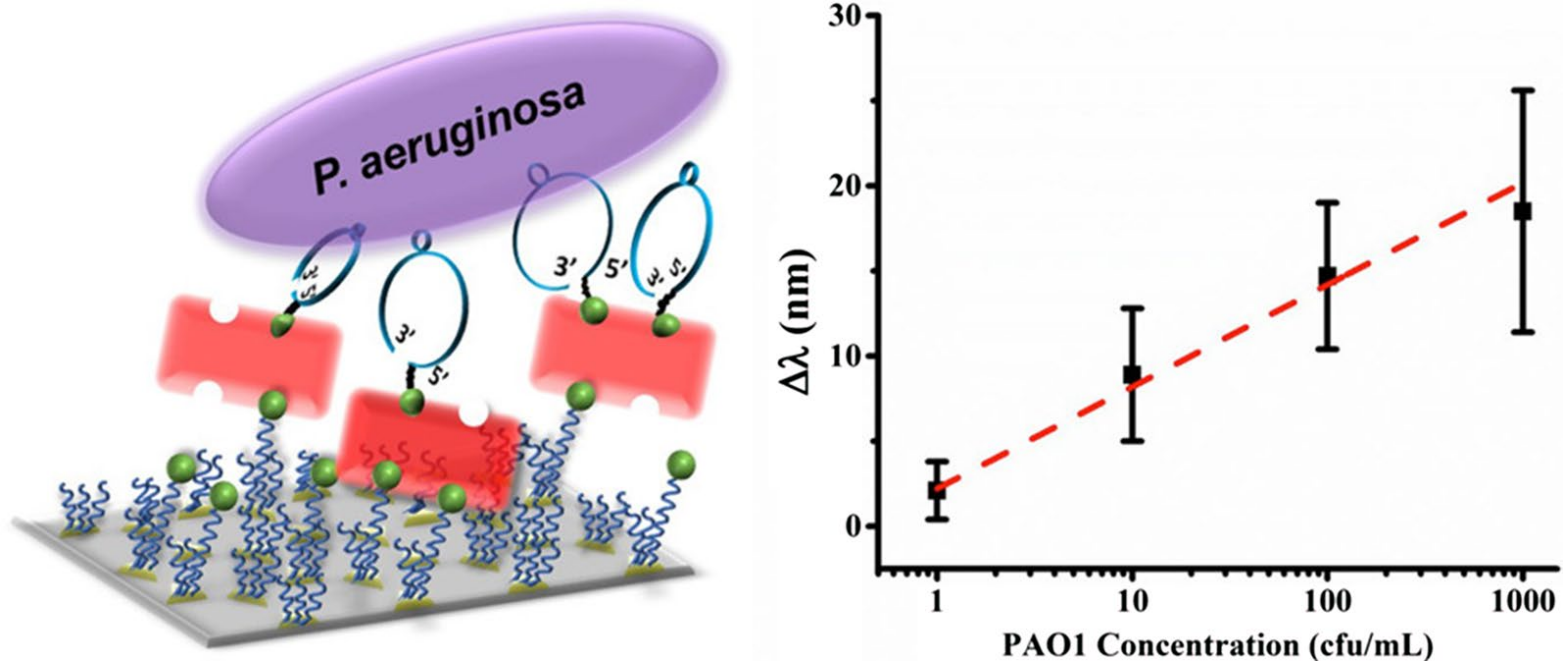
Schematic of *P. aeruginosa* LSPR sensor chip (left). Sensor calibration curve, where error bar represents the standard deviation of all data points at a specific bacterial concentration (right). (Reprinted with permission from Hu et al. [144]. Copyright 2018 American Chemical Society)

The first *P. aeruginosa* aptamer was discovered by Wang et al. [148] in 2011 and subsequently has been used in a range of sensors. The discussion that follows highlights two sensors that utilize optical transduction. Yoo et al. [142] and Hu et al. [144] fabricated nano-textured substrates to produce localized surface plasmon resonance (LSPR) chips (Fig. 7). Yoo et al. choose a three-step fabrication approach, first gold was deposited on a glass slide, silica nanoparticles were then deposited and then followed by the deposition of a second gold layer whereas Hu et al. opted for standard nanosphere lithography. The two groups also chose different methods to functionalize the sensor with Yoo et al. attaching the aptamers directly to the sensor surface via a gold-thiol bond. In contrast, Hu et al. used a polyethylene glycol (PEG) spacer to minimize steric hindrance for the aptamers with the goal of achieving a lower detection level. Hu et al. were successful at developing a sensor with a linear response at low concentrations and a lower limit of detection, 10 CFU mL^−1^ vs. Yoo et al.’s 10^4^ CFU mL^−1^. It should be noted that one of Yoo et al.’s goals was to create a low volume sensor and that their LOD was obtained in a 3 µL sample.

## Conclusions

Nanosensor development for environmental contaminants is growing rapidly and, as described throughout this review, nanomaterials and recognition agents are continuously being combined in new and creative ways. The recent developments in sensor design aim to overcome the shortcomings of first-generation sensors such as nonspecific binding, particle size variation, nanoparticle aggregation, and nanoparticle stability. Questions of assay selectivity and sensitive in complex environmental matrices remains but a growing number of reports are using representative matrices to demonstrate the stability and selectivity of their sensors. The robustness of field deployable sensors is a must if individuals are going to be empowered to analyze their environment.

## Abbreviations

Abs: antibodies
AChE: acetylcholinesterase
AgNP: silver nanoparticles
APDC: ammonium pyrrolidine dithiocarbamate
AuNP or GNP: gold nanoparticles
AuNP/BC: gold nanoparticle/bacteria cellulose
BSA: bovine serum albumin
Cd: cadmium
CFU: colony-forming unit
CNT: carbon nanotubes
Cr: chromium
CS: chitosan
CT: cholera toxin
DNA: deoxyribonucleic acid
dNTP: deoxyribonucleotide triphosphate
DPV: differential pulse voltammetry
Fe_3_O_4_: magnetite
FRET: fluorescence resonance energy transfer
FTIC: fluorescein isothiocyanate
GCE: glassy carbon electrodes
Hg: mercury
ICA: immunochromatographic assay
LOD: fluorescence resonance energy transfer
LSPR: localized surface plasmon resonance
mAbs or McAb: monoclonal antibodies
MB: magnetic bead
MNP: magnetic nanoparticles
MPA: 3-mercaptopropionic acid
mRNA: messenger RNA
NMO: nanostructured metal oxides
NP: nanoparticles
OP: organophosphorus pesticide
pAbs: polyclonal
Pb: lead
PCR: polymerase chain reaction
PEG: polyethylene glycol
PEI: polyethylenimine
PL: photoluminescence
PPy: polypyrrole
QD: quantum dot
R6G: rhodamine 6G
rA: ribonucleoside adenosine
RCA: rolling circle amplification
rGO: reduced graphene oxide
RNA: ribonucleic acid
S: sulfur
Se: selenium
SEF: surface enhanced fluorescence
SELEX: systematic evolution of ligands by exponential enrichment
SERS: surface enhanced Raman spectroscopy
SPR: surface plasmon resonance
SPRi: surface plasmon resonance imaging
ssDNA: single stranded DNA
SWASV: square wave anodic stripping voltammetry
SWCNT: single wall carbon nanotubes
Te: tellurium
TGA: thioglycolic acid
TiO_2_: titanium dioxide
TMB: 3,3,5,5-tetramethylbenzidine
WHO: World Health Organization
Zn: zinc
γ-Fe_3_O_4_: maghemite
